# FioSchisto’s expert perspective on implementing WHO guidelines for schistosomiasis control and transmission elimination in Brazil

**DOI:** 10.3389/fimmu.2023.1268998

**Published:** 2023-12-05

**Authors:** Camilla Almeida Menezes, Langia Colli Montresor, Soraya Torres Gaze Jangola, Aline Carvalho de Mattos, Ana Lúcia Coutinho Domingues, Arnaldo Maldonado Júnior, Clélia Christina Mello Silva, Constança Simões Barbosa, Cristiane Lafetá Furtado de Mendonça, Cristiano Lara Massara, Cristina Toscano Fonseca, Edward José de Oliveira, Elainne Christine de Souza Gomes, Elizângela Feitosa da Silva, Fernando Schemelzer de Moraes Bezerra, Floriano Paes Silva-Jr, Isadora Cristina de Siqueira, José Roberto Machado e Silva, Leo Heller, Leonardo Paiva Farias, Lilian C. Nobrega Holsbach Beck, Mariana Cristina Silva Santos, Mariana Gomes Lima, Marina de Moraes Mourão, Martin Johannes Enk, Monica Ammon Fernandez, Naftale Katz, Omar dos Santos Carvalho, Patrícia Martins Parreiras, Renata Heisler Neves, Sandra Grossi Gava, Sheilla Andrade de Oliveira, Silvana Carvalho Thiengo, Tereza Cristina Favre, Carlos Graeff-Teixeira, Otávio Sarmento Pieri, Roberta Lima Caldeira, Rosiane A. da Silva-Pereira, Roberto Sena Rocha, Ricardo Riccio Oliveira

**Affiliations:** ^1^ Instituto Gonçalo Moniz, Fundação Oswaldo Cruz - FIOCRUZ, Salvador, Brazil; ^2^ Instituto René Rachou, Fundação Oswaldo Cruz - FIOCRUZ, Belo Horizonte, Brazil; ^3^ Instituto Oswaldo Cruz, Fundação Oswaldo Cruz - FIOCRUZ, Rio de Janeiro, Brazil; ^4^ Centro de Ciências da Saúde, Departamento de Medicina Clínica, Universidade Federal de Pernambuco, Recife, Brazil; ^5^ Instituto Aggeu Magalhães, Fundação Oswaldo Cruz - FIOCRUZ, Recife, Brazil; ^6^ Centro de Ciências da Saúde, Departamento de Análises Clínicas e Toxicológicas, Universidade Federal do Ceará, Fortaleza, Brazil; ^7^ Faculdade de Ciências Médicas, Universidade Estatual do Rio de Janeiro, Rio de Janeiro, Brazil; ^8^ Seção de Parasitologia, Instituto Evandro Chagas, Ananindeua, Brazil; ^9^ Centro de Ciências da Saúde, Núcleo de Doenças Infecciosas, Universidade Federal do Espírito Santo, Vitória, Brazil

**Keywords:** Schistosoma mansoni, schistosomiasis, neglected tropical diseases, control and elimination, Brazil

## Abstract

The World Health Organization (WHO) recognizes schistosomiasis as one of the Neglected Tropical Diseases targeted for global elimination in the 2030 Agenda of the Sustainable Development Goals. In Brazil, schistosomiasis mansoni is considered a public health problem, particularly prevalent among vulnerable populations living in areas with poor environmental and sanitary conditions. In 2022, the WHO published a Guideline encompassing recommendations to assist national programs in endemic countries in achieving morbidity control, eliminating schistosomiasis as a public health problem, and advancing towards interrupting transmission. The perspectives presented here, collectively prepared by members of the Oswaldo Cruz Foundation’s (Fiocruz) Schistosomiasis Translational Program (FioSchisto), along with invited experts, examine the feasibility of the WHO recommendations for the Brazilian settings, providing appropriate recommendations for public health policies applicable to the epidemiological reality of Brazil, and suggests future research to address relevant issues. In Brazil, the provision of safe water and sanitation should be the key action to achieve schistosomiasis elimination goals. The agencies involved in measures implementation should act together with the Primary Care teams for planning, executing, monitoring, and evaluating actions in priority municipalities based on their epidemiological indicators. Host snails control should prioritize judicious ecological interventions at breeding sites. The Information, Education, and Communication (IEC) strategy should be associated with water and sanitation and other control actions, actively involving school community. To identify infected carriers, FioSchisto recommends a two-stage approach of immunological and molecular tests to verify transmission interruption during the intervention and beyond. Praziquantel administration should be done under medical supervision at the Primary Care level. MDA should be considered in exceptional settings, as a measure of initial attack strategy in locations presenting high endemicity, always integrated with water and sanitation, IEC, and snail control. To assist decision-making, as well as the monitoring and evaluation of strategic actions, there is a need for an Information System. FioSchisto considers this systematization essential to make investments in strategic research to support the improvement of schistosomiasis control actions. Efforts toward schistosomiasis elimination in Brazil will succeed with a paradigm shift from the vertical prescriptive framework to a community-centered approach involving intersectoral and interdisciplinary collaboration.

## 1 Introduction

Schistosomiasis, one of the oldest parasitic infections in humans, has co-evolved with humanity over centuries. Currently, an estimated 250 million people worldwide are infected with the disease, while approximately 700 million people are at risk of infection. Schistosomiasis has been recognized as one of the Neglected Tropical Diseases (NTDs), caused by blood flukes such as *Schistosoma mansoni*, *S. haematobium*, and *S. japonicum*. The disease primarily affects impoverished regions and individuals, remaining endemic in 78 countries (1). It is acquired through the skin and mucous membrane contact with water containing the infective forms of the parasite and is more prevalent in areas lacking adequate water and sanitation (2).

The transmission of schistosomiasis relies on infected individuals excreting helminth eggs within their feces and urine and aquatic snails acting as intermediate hosts that release infective cercariae into water sources used by humans in their daily activities. Schistosomiasis transmission is a complex process influenced by numerous complexes contributing factors. As a result, controlling the disease requires implementing multiple preventive measures. These measures include early diagnosis and timely treatment, the monitoring and control of intermediate hosts, health educational initiatives, and sanitation efforts to modify the environmental conditions that facilitate *Schistosoma* spp. transmission. These actions must be implemented in a coordinated and integrated manner as part of a comprehensive control program (2).

Align with the United Nations’ 2030 agenda established in 2015, the World Health Organization (WHO) has set a goal of eliminating schistosomiasis as a public health issue worldwide. The aim is to reduce the prevalence of severe infections (≥ 400 eggs per gram of feces) to less than 1% by 2030 and halt transmission in at least 25 out of the 78 endemic countries during the same period. To achieve these goals, the WHO published in 2022 guidelines for the control and elimination of human schistosomiasis. These guidelines provide a set of recommendations to guide national schistosomiasis programs in endemic countries to achieve morbidity control, elimination of the disease as a public health issue, and to make progress toward interrupting transmission. WHO guidelines prioritize mass drug administration (MDA) while also advocating for sustainable preventive measures such as water and sanitation (1).

Research conducted in Brazil, where schistosomiasis mansoni is a significant public health concern, has shown that periodic mass treatment of people living in endemic communities if implemented without concurrent improvements in water supply and sanitation infrastructure resulted in temporary impact in the reduction of disease prevalence (3). In light of this, the present discussion, collaboratively conducted by members of the Oswaldo Cruz Foundation’s Schistosomiasis Translational Program (FioSchisto) and invited experts, explores the feasibility of implementing WHO recommendations for Brazilian settings. The outcome is a set of recommendations for public health policies that apply to the country’s epidemiological context and suggests pertinent research topics to be addressed.

## 2 Analysis of WHO recommendations for schistosomiasis control and transmission elimination

This section will discuss the six WHO recommendations for the control and elimination of schistosomiasis by 2030 (1), as shown in **Table 1**, from the perspective of the Brazilian context, where low endemicity areas are widespread and *S. mansoni* is the only species occurring.

**Table 1:** WHO recommendations for schistosomiasis control and elimination.

Recommendation 1	
In endemic communities with a prevalence of *Schistosoma* spp. infection ≥ 10%, WHO recommends annual preventive chemotherapy with a single dose of praziquantel at ≥ 75% treatment coverage in all age groups from 2 years old, including adults, pregnant women after the first trimester, and lactating women, to control schistosomiasis morbidity and advance towards eliminating the disease as a public health problem.	
Strong recommendation	Certainty of evidence: moderate
Recommendation 2	
In endemic communities with a prevalence of *Schistosoma* spp. infection < 10%, WHO suggests one of two approaches based on programmatic objectives and resources: (i) where there has been a program of regular preventive chemotherapy, to continue the intervention at the same or reduced frequency towards interruption of transmission; or (ii) where there has not been a program of regular preventive chemotherapy, to use a clinical approach of test-and-treat, instead of preventive chemotherapy targeting a population.	
Conditional recommendation	Certainty of evidence: very low
Recommendation 3	
In endemic communities with a prevalence of *Schistosoma* spp. infection ≥ 10% that demonstrate a lack of an appropriate response to annual preventive chemotherapy, despite adequate treatment coverage (≥ 75%), WHO suggests consideration of biannual (twice yearly) instead of annual preventive chemotherapy.	
Conditional recommendation	Certainty of evidence: very low
Recommendation 4	
WHO recommends that health facilities provide access to treatment with praziquantel to control morbidity due to schistosomiasis in all infected individuals regardless of age, including infected pregnant excluding the first trimester, lactating women, and pre-SAC aged < 2 years. The decision to administer treatment in children under 2 years of age should be based on testing and clinical judgment.	
Strong recommendation	Certainty of evidence: moderate
Recommendation 5	
WHO recommends WASH interventions, environmental interventions (water engineering and focal snail control with molluscicides), and behavioral change interventions as essential measures to help reduce transmission of *Schistosoma* spp. in endemic areas.	
Strong recommendation	Certainty of evidence: low
Recommendation 6	
In communities approaching the interruption of transmission (defined as having no autochthonous human cases reported for 5 consecutive years), WHO suggests a verification framework that consists of: 1. Testing for *Schistosoma* infection in humans with a diagnostic that has high sensitivity and specificity. This may require the use of a two-step diagnostic process starting with a high-sensitivity test confirmed with a second, high-specificity test. 2. Testing for *Schistosoma* infection in snails with a diagnostic that has high sensitivity and specificity. This may require the use of a two-step diagnostic process starting with a high-sensitivity test confirmed with a second, high-specificity test. 3. Testing for *Schistosoma* infection in non-human mammalian hosts, as applicable, with a diagnostic that has high sensitivity and specificity. This may require the use of a two-step diagnostic process starting with a high-sensitivity test confirmed with a second, high-specificity test.	
Conditional recommendation	Certainty of evidence: low

### 2.1 Analysis of recommendations 1, 2, and 3

#### 2.1.1 Background

The first three WHO’s recommendations for the control and elimination of schistosomiasis emphasize the strategy of MDA using praziquantel in different epidemiological scenarios. Some challenges and criticisms need to be considered regarding these recommendations. The first challenge relates to the possibility of an insufficient stock of medication available to meet global demand. As for the criticisms, there is scientific evidence suggesting that praziquantel mass administration may only provide temporary benefits.

Studies conducted in Brazil have demonstrated that periodic treatment of endemic communities without adequate water supply and sanitation has a transient effect. Examples of this scenario occurred in some locations in the municipalities of Ipojuca in Pernambuco and Conde in Bahia, which maintains the endemicity of the disease after successive mass treatments over two decades (4–8).

In 2009, the Executive Council of the Pan American Health Organization (PAHO) reiterated the WHO recommendation to implement an annual MDA scheme targeting school-age children in endemic areas of the Americas. At that time, the Brazilian Ministry of Health argued that schistosomiasis control measures should focus on strengthening the diagnosis and treatment capabilities at the primary healthcare level, as well as improving sanitation conditions (9). According to the Ministry of Health’s latest Health Surveillance Guide (10), several crucial measures need to be taken to attain the required health standards for safeguarding and enhancing the living conditions of at-risk populations vulnerable to schistosomiasis and other infectious diseases that aggravate poverty. These measures include ensuring access to safe drinking water and sanitation, managing solid waste disposal, adopting appropriate practices for the use of land, improving drainage, controlling vectors, and managing non-human reservoirs.

Since 2014, the Ministry of Health has limited its recommendation of MDA to communities where the Kato-Katz positivity rates are ≥ 25%, which are a small minority, and always in conjunction with the aforementioned genuinely preventive measures (2). In Brazil, MDA has been implemented only once in communities where Kato-Katz positivity rates were 10% or higher. This initiative took place in different locations considered a priority by the Pernambuco State Health Department, in the four-year periods of 2011-2014 (11), 2015-2018 (12) e 2019-2022 (13). Despite the implementation of MDA, the failure to improve environmental sanitation and provide clean water to the communities left the population at a persistent risk of reinfection (14, 15).

A recent publication considers that the Americas and Asia may have had already achieved the elimination of schistosomiasis as a public health problem and are heading toward the interruption of transmission (16). This consideration should be viewed with caution regarding Brazil, as it has not yet been validated that the proportion of exams with severe infections (≥ 400 epg by Kato-Katz) in the country is lower than 1%, which is a prerequisite stipulated by the WHO (1). According to data from the Surveillance and Control Program for Schistosomiasis (SISPCE) (2), in 2016, 9% of the 53 localities that underwent MDA in the state of Pernambuco sustained percentages of severe infections equal to or higher than 1%.

In 2018, the Brazilian Ministry of Health formulated an Action Plan to tackle schistosomiasis for the period 2019-2021. For this, 472 municipalities across 11 states were chosen based on epidemiological data from official information systems over the previous five years, as well as results from the National Survey of Schistosomiasis and Soil-transmitted Helminthiasis (INPEG) conducted between 2011 and 2015. Factors such as population data, Municipal Human Development Index, and water and sanitation conditions of the municipalities in the period 2017-2018 were also considered. However, the original plan was not implemented due to the COVID-19 pandemic. The Ministry of Health is developing a new version, taking into account the goal of eliminating schistosomiasis as a public health problem by 2030 (1) and incorporating the latest recommendations provided by the WHO (17).

It is expected that the goal of eliminating schistosomiasis as a public health problem in Brazil will be achieved in the coming years, leading to an official recommendation by the WHO to proceed towards control and elimination of transmission. This new stage will require the implementation of a test-and-treat scheme dependent on highly sensitive and specific diagnostic tests. Consequently, both MDA and the identification of infected individuals based on Kato-Katz (18) will no longer be applicable. To achieve the goal of interrupting transmission, it will be crucial to prioritize the preventive measures emphasized in the Ministry of Health’s Health Surveillance Guide (10).

#### 2.1.2 Recommendations from FioSchisto

FioSchisto understands that the WHO recommendations regarding the mass administration of praziquantel do not apply to the current eco-epidemiological context of Brazil. Instead, interventions should genuinely prioritize preventive measures in both localities that have not yet achieved the goal of eliminating schistosomiasis as a public health problem and those moving towards interrupting transmission. Furthermore, municipalities interested in validating the goal of eliminating schistosomiasis as a public health problem should first identify the target communities, as these are the operational units of actions for schistosomiasis control (1, 2, 17), not the municipalities.

The FioSchisto highlights the need for improved information systems to assist in the decision-making process regarding the recommended strategic actions for the elimination of schistosomiasis as a public health problem and the interruption of transmission, as well as the monitoring and evaluation of these actions. The existing system (SISPCE) is deemed inadequate in terms of its ability to register, consolidate, and provide the required information. A robust information system should encompass data at both the individual and target population levels throughout the baseline, intervention, and follow-up stages.

Although FioSchisto acknowledges that MDA may not universally apply to the current Brazilian epidemiological settings, we recognize that there are still areas with a high frequency of positive parasitological tests. For such areas, the local health system and respective health authorities must mobilize attention to establish an effective strategy for local action, prioritizing actions based at water supply, sanitation, and health education, and may consider MDA as an initial attack strategy.

#### 2.1.3 Investment in research

The FioSchisto recognizes that the 10% positivity threshold established by WHO for Kato-Katz testing applies specifically to different MDA schemes and not to other interventions. These interventions are indicated regardless of the level of positivity observed in the communities. It is worth noting that the threshold defined by WHO for MDA is based on studies conducted in Sub-Saharan Africa, where the epidemiological conditions differ from those in Brazil. Thus, the determination of appropriate cut-off points for different intervention schemes depends on adequate studies of the country’s reality. The FioSchisto emphasizes the need for investment in research to better understand the epidemiological context and to guide the development of appropriate interventions for the Brazilian context.

### 2.2 Analysis of recommendation 4

#### 2.2.1 Background

The fourth WHO recommendation emphasizes that the use of praziquantel is indicated for all infected individuals, with exceptions for pregnant women in the first trimester, breastfeeding women, and children under two years of age. It also states that access to treatment should be provided by healthcare services in adequate facilities.

The praziquantel leaflet, approved by the Brazilian National Health Surveillance Agency (ANVISA), states that the treatment is not recommended for children under four years of age. Similarly, praziquantel treatment is contraindicated in the first trimester of pregnancy. However, due to the lack of scientific evidence on the safety of praziquantel use during pregnancy, treatment for pregnant women from the second trimester onwards should only be carried out after rigorous medical evaluation. In this case, praziquantel administration is classified as a category B risk.

According to ANVISA, during lactation, the prescribing physician should also carefully evaluate the administration of praziquantel since the medication can reach breast milk in concentrations equivalent to up to 20% of plasma concentration. In case of indication and use of praziquantel during lactation, breastfeeding should be interrupted on the day of treatment and during the three following days after administration.

#### 2.2.2 Recommendations from FioSchisto

FioSchisto considers that praziquantel can be administered to patients under the mentioned conditions, but it should be done under careful medical evaluation and supervision. However, the indication and administration of praziquantel for children under four years of age, pregnant women, or lactating women should be implemented on an individual basis and in appropriate medical facilities, which does not apply at the population or large-scale level.

Additionally, FioSchisto recommends that primary healthcare teams in each municipality should be in charge of the schistosomiasis treatment using praziquantel. Thus, it is recommended that this team is properly trained and capacitated in the treatment of schistosomiasis. It is the responsibility of each municipality to provide the necessary support and resources to enable the implementation of this training.

#### 2.2.3 Investment in research

Considering that praziquantel is currently the only available medication for treating schistosomiasis, investment in research for the development of new drugs is necessary. In addition to that, the well-known low efficacy against immature forms of *Schistosoma* sp., the continuous use of the medication for over four decades, and the parasite’s ability to develop resistance to praziquantel make it necessary to develop new therapeutic approaches. Furthermore, it should be noted that the current formulation of praziquantel is not recommended for children under four years of age, according to the medication’s package insert.

The pediatric praziquantel, which is being produced by Farmanguinhos-Fiocruz through a Pediatric Praziquantel consortium initiative led by Merck, will be available in the short term. This will be an important advancement in the treatment of schistosomiasis for children under four years of age, who have been historically excluded from therapeutic interventions. However, since the clinical trial for this new drug has not been conducted in Brazil and considering the large variation among human and parasite populations, it is recommended that its safety and efficacy should be also demonstrated in the Brazilian settings.

### 2.3 Analysis of recommendation 5

#### 2.3.1 Background

The fifth WHO recommendation focuses on WASH interventions and chemical control of snails. The term WASH stands for Water, Sanitation, and Hygiene. However, the document introduces these interventions as of low certainty of evidence and states that “WASH interventions are expected to provide modest benefits in limiting Schistosoma transmission”. For WHO, WASH interventions should be a “complementary measure” to reduce the prevalence of schistosomiasis.

As there is no exact translation of this term in the technical language used in Brazil, the expression “basic sanitation” is considered for this purpose. According to Brazilian legislation (19), basic sanitation comprises actions related to water supply, sanitation, solid waste management, and drainage of rainwater. Therefore, the concept of “basic sanitation” is more comprehensive than the actions covered by the WASH expression. In this study, the term “basic sanitation” is used from this point on, considering the above-mentioned conceptual difference.

While the inequality in access to sanitation infrastructure poses a hindrance to achieving universalization, there have been notable advances in this field in Brazil. Since the first national prevalence surveys in the 1950s, the country has gone from 15.5% to 86% coverage of households with water supply in 2019. For sanitation services, Brazil went from 9% of households served to 68% during the period (20). This increase represents significant additions in access to sanitary infrastructure over the decades, which may be compatible with efforts toward fulfilling Human Rights in Water and Sanitation (21).

Brazil has been consistently developing models to control schistosomiasis that can substantially reduce the prevalence and incidence indicators of the disease. The strategies include drug treatment, provision of water, sanitation and hygiene facilities, and actions that involve community participation and primary healthcare within the Unified Health System (SUS).

Studies conducted in Brazil have shown the effectiveness of basic sanitation interventions in reducing infection rates. For example, in the 1960s, in a locality in the municipality of Cabo, in Pernambuco, infection rates were progressively reduced in areas with basic sanitation interventions compared to areas without intervention (22). Also in Pernambuco, in the municipality of São Lourenço da Mata, sanitary facilities such as septic tanks (OR 0.60; 95% CI 0.44-0.84) and general sewage systems (OR 0.20; 95% CI 0.14-0.29) were significantly associated with a decrease in the probability of infection (23). Another study, conducted in 482 municipalities in the state of Minas Gerais, showed that the variable “percentage of households with general sewage systems” contributed as an explanatory factor for infection rates, along with other social, demographic, and health conditions evaluated in the population. Additionally, the variable “percentage of households with sewage discharged into rivers, lakes, or seas” provided indicative evidence for the occurrence of the disease (24).

Similarly, an experience in municipalities of the states of Espírito Santo and Minas Gerais demonstrated that providing dry toilets and water, as well as facilities that encourage hygiene practices by the population, such as community laundries, domestic washing tanks, drinking fountains, faucets, and showers, constituted relevant alternatives to individualized drug treatment (25, 26). A district in the municipality of Sabará, Minas Gerais, showed a positive response to the implementation of a treated water system between 1980 and 2007, with a reduction in the prevalence of schistosomiasis from 36.7% to 2.5% (27, 28). In this state, studies in an endemic area over 25 years showed that severe forms of the disease were strongly associated with the absence of piped water (OR 7.7; 95% CI 2.6-23.1) and the habit of bathing in water collections (OR 5.7; 95% CI 1.3-25.5), the latter being a consequence of the former. In the observed periods (1981, 1992, and 2005), water supply coverage increased from 33.7% to 96%, safe sanitation increased from 71.7% to 97.6%, and the prevalence of the disease decreased from 70.4% to 1.7% (29–31).

The effectiveness of interventions in the school environment, compared to actions in the community, has been reported and should be considered. An ecological study using prevalence data from the INPEG (32) and national household and public and private school sanitation data showed that access to safe drinking water in schools was a protective factor for the disease (PRR 0.982; 95% CI 0.970 - 0.995) (33). This reinforces that water contact patterns are influenced by the availability of safe water supply, sanitation, and health education (34–36).

From a historical perspective, an ecological study analyzed data from three national surveys of schistosomiasis (1947-1953, 1975-1979, and 2010-2015), with a sample of 1,721 municipalities and 1,182,339 schoolchildren aged 7-14. The study found a protective effect of access to sanitary sewerage (RR 0.996; 95% CI 0.994 - 0.998), that increased from a mean coverage of 2.6% to 30.6% in the studied municipalities over 70 years. This result suggests that interventions in sanitation at the collective level have the potential to insert an effective barrier in the transmission cycle of schistosomiasis (37).

The local and nationwide findings reaffirm international studies that emphasize the increase in access to clean water, sanitation, and hygiene practices as important measures to reduce the chances of infection by the parasite (38). A meta-analysis published in 2014 supports that safe water supply (OR 0.53; 95% CI 0.47 - 0.61) and sanitation (OR 0.59; 95% CI 0.47 - 0.73) are associated with significantly lower chances of occurrence of schistosomiasis (39). It is important to highlight that this publication included 44 relevant studies, with Brazil contributing with 15 studies for water data and 11 studies for sanitation, reinforcing the country’s leadership in research on the topic.

Regarding the chemical control of snails, during the 20th century, the use of molluscicides was among the strategies most advocated by governments and health agents (40–42). Since the 1960s, the most commonly used molluscicide has been niclosamide (40). In theory, the lethal concentration for snails, considered low, is non-toxic for vertebrates, including fish and humans. However, due to the technical complexity of the process of applying molluscicides in the environment, their irregular dispersion can lead to a higher accumulation in some areas, which can cause fish mortality and health problems in humans (43). Moreover, *Biomphalaria* sp. is known to undergo natural dormancy in certain periods, resulting in the inefficacy of niclosamide application.

Some countries, such as Brazil, have imposed restrictions on the use of niclosamide in the environment due to concerns regarding its harmful effects on non-target organisms (44). Niclosamide was approved many decades ago for anticestodal treatment in humans and has recently been evaluated in anticancer therapy and the treatment of the Zika virus (45, 46). This demonstrates its action on various signaling pathways in humans and other organisms, underscoring the need for a comprehensive risk evaluation of its environmental use. Furthermore, the decrease in the cost of praziquantel, after the end of its patent in the early 1990s, has made the use of niclosamide less cost-effective and potentially obsolete for schistosomiasis control.

Considering the evidence on the importance of long-lasting measures for snail control in the management of schistosomiasis, the promotion of environmental modifications that contribute to the reduction of snail populations, such as the modification of natural habitats, has been encouraged. Countries such as Japan, Morocco, Saudi Arabia, and Venezuela have achieved success with strategies such as the removal of aquatic vegetation, drainage of flooded areas, the lining of irrigation canals, and modification of watercourses, with careful attention to minimizing undesirable environmental impacts (47–50). In contrast, in areas with schistosomiasis transmission, environmental modifications that potentially contribute to the increase and expansion of snail populations, such as dam construction and expansion of irrigation, should be avoided (51, 52).

#### 2.3.2 Recommendations from FioSchisto

In contrast to the WHO, FioSchisto proposes that genuinely preventive and long-lasting interventions against schistosomiasis should be prioritized in Brazil. It includes basic sanitation, health education, and interventions in snail breeding sites. The group recommends that a more appropriate approach should consider the following key aspects: the current public policy in Brazil based on decentralized control of endemic diseases; the autonomy of primary health care in addressing local health issues; the One Health concept; and environmental control measures of the disease, including interventions in basic sanitation.

The main basic sanitation measures that should be considered for schistosomiasis control in the country, after a thorough evaluation of the socio-environmental conditions of the affected locality, are described in **Table 2**.

**Table 2:** Main sanitation measures to be considered for schistosomiasis control in Brazil.

Basic sanitation measures
Supply of water for human consumption, with sufficient quantity and quality that meets potability standards.
Sanitary sewage disposal, with static or dynamic solutions, providing adequate sewage treatment to control pathogens and whose operation and maintenance are within reach of the community and service providers
Improvement of intra-household sanitary conditions.
Collection and proper disposal of solid waste, when relevant.
Drainage of rainwater, when applicable.

In selecting the localities to prioritize basic sanitation interventions, it is important to adopt a watershed perspective. So it may be necessary to implement actions not only in endemic areas but also in contiguous areas that share the same watershed and are epidemiologically significant. In situations where there are increased migratory flows, due to development projects such as irrigation, dams, and mining works, basic sanitation actions must be properly planned and simultaneously implemented. In these cases, the risk of the introduction of schistosomiasis should be evaluated, and the necessary preventive environmental measures should be implemented.

It is recommended to establish effective and permanent communication between federal agencies and municipal health and environment departments to discuss, evaluate, plan, and implement sanitary interventions. Structural implementations should consider the sanitary reality of each region, given that the country has highly heterogeneous environmental, epidemiological, and socioeconomic conditions. The primary criterion for prioritizing interventions must be the prevalence of schistosomiasis and other sanitation-reducible diseases.

Health promotion, disease prevention, and control actions should be developed at the municipal level, taking into account local peculiarities. It is important to recognize that behavioral changes cannot be imposed through top-down interventions alone. Instead, it should be adapted through the understanding of risk factors and cultural patterns and in association with structural changes in the living environment.

Health professionals, including trained Community Health Agents, are essential and should be always included in the development of activities to inform and promote discussions about the disease, and the factors involved in transmission, prevention, and control. These actions should occur in the community and the school environment, as children and young people, are particularly important in the parasite transmission cycle. These discussions provide an opportunity to address that access to water and sanitation is a fundamental human right and a responsibility of public services, transcending beyond the scope of the disease itself.

Regarding intervention in snail breeding sites, FioSchisto does not recommend the use of molluscicides, such as niclosamide, for snail control in Brazil due to its toxicity. Whenever possible, it is recommended to use snail control alternatives that are more appropriate and aligned with the One Health concept, particularly in low-endemicity areas, which are predominant in Brazil.

The FioSchisto suggests maintaining the recommendations outlined in the Technical Guidelines of the Ministry of Health (53) for snail control, with some updates and adjustments. As physical control methods in snail breeding sites, environmental management is considered, based on measures such as the rectification, coating, or channeling of watercourses, aimed at increasing the speed of water and making it difficult for intermediate hosts to attach. These measures should be planned and implemented in a way to minimize environmental impacts. Periodic cleaning and removal of aquatic vegetation on the banks of watercourses should be considered as a form of maintenance. Another recommended measure is the filling or drainage of places where water accumulates and creates an environment favorable to snail proliferation. In situations where contact with watercourses is necessary for crossing, the construction of bridges should be considered.

### 2.3.3 Investment in research

Investments in research are essential, especially in the areas of environmental, biological, and chemical control of intermediate hosts.

Regarding environmental control, there is still a need for a better understanding of the effects of implementing basic sanitation on the control of schistosomiasis and other parasitic diseases. From one side, basic sanitation encompasses a set of possible technologies and interventions (among other variations, collective vs individual systems for water; onsite vs offsite solutions for sanitation). On the other, the epidemiological, environmental, and socioeconomic context influences the possible effects of these interventions. This poses the need for fine-tuning in the research of the effects of different solutions for basic sanitation in different contexts, about the control of schistosomiasis.

Regarding biological control, there is a need to investigate the use of other native species within the same hydrographic basin proving it is sustainable and free of undesirable environmental effects. This approach can reduce or eliminate populations of schistosomiasis-transmitting snails. Another alternative is the release of snail populations resistant to *S. mansoni* infection in transmission foci. So, through crossbreeding with susceptible local populations, the trait of resistance or reduced susceptibility can be disseminated. It is strongly encouraged that these studies be carried out on populations of *Biomphalaria glabrata*, the main intermediate host of *S. mansoni* in Brazil. Also, the use of post-genomic methodologies for genetic modification of intermediate hosts to promote castration or resistance to *S. mansoni* infection shows promise.

Regarding chemical control, the focus should be on the development of solutions with high specificity and low environmental impact. It is suggested to search for new chemical products with molluscicidal or schistosomicidal action that are specific and target essential molecular targets for the survival and development of the snail and/or the parasite.

### 2.4 Analysis of recommendation 6

#### 2.4.1 Background

The sixth and final WHO’s recommendation addresses a strategy for confirming the interruption of schistosomiasis transmission in communities, defined as the absence of autochthonous cases reported in humans in a specific community for five consecutive years. This recommendation suggests testing for schistosomiasis infection in humans, snails, and other mammalian hosts.

Regarding the verification of the interruption of transmission, in the absence of sensitive monitoring tools, there is a risk of premature interruption of the controlling measurement, which can lead to a significant increase in the number of cases in a short period of time. Therefore, the use of an accurate test for the diagnosis of schistosomiasis is important not only for the certification of disease elimination, but throughout the intervention period, especially when the control actions are guided by diagnosis performed at individual level and guides control actions. It is worth mentioning that the quantification of the parasitic load is necessary to indicate the elimination of the disease as a public health problem. But, considering the reality of the Brazilian health systems, except Kato-Katz, no other diagnostic methods are currently available for quantification of the parasitic load.

For the diagnosis of *S. mansoni* infection in the intermediate hosts, the main limitation is related to the transportation of the mollusks. Brazilian legislation is restrictive for the transport of biological material, turning the sample transport difficult and the mollusk-monitoring unfeasible. In addition to the high shipping fees, the time elapsed between collection and shipment of biological material is too long, resulting in the death of snails and in the waste of human and financial resources used in the process, making monitoring efforts unviable.

#### 2.4.2 Recommendations from FioSchisto

FioSchisto considers that population surveys seeking the identification of infected individuals should use diagnostic methods with sensitivity greater than that achieved by the analysis of two slides of Kato-Katz. Thus, for areas that have not achieved the status of interrupting transmission, the group recommends the use of commercial serological tests. It is known that the currently available serological tests registered at ANVISA have limitations in terms of specificity. This limitation can be compensated by the epidemiological profile of the endemic areas, resulting in a high positive predictive value of the test in areas with high infection prevalence. Additionally, it should be noted that ELISA tests for other infections are routinely performed, and the operational capacity for conducting these tests already exists.

For localities in the process of eliminating transmission, where the predictive values of serological tests are compromised given the low incidence of the disease, and since no single diagnostic test is capable of accurately detect schistosomiasis in this epidemiological scenario, FioSchisto endorses the two-step diagnostic strategy recommended by WHO. It is suggested that the first screening stage should be performed using commercial serological tests with high sensitivity and the second stage should use a molecular test, such as quantitative Polymerase Chain Reaction (qPCR) (54), which, despite being more complex, have higher sensitivity compared to Kato-Katz method and a higher level of specificity. It is worth noting that the COVID-19 pandemic has shown that Brazil has the infrastructure needed to perform molecular tests, improving diagnosis and reducing costs if implemented on a larger scale.

**Figure 1:**
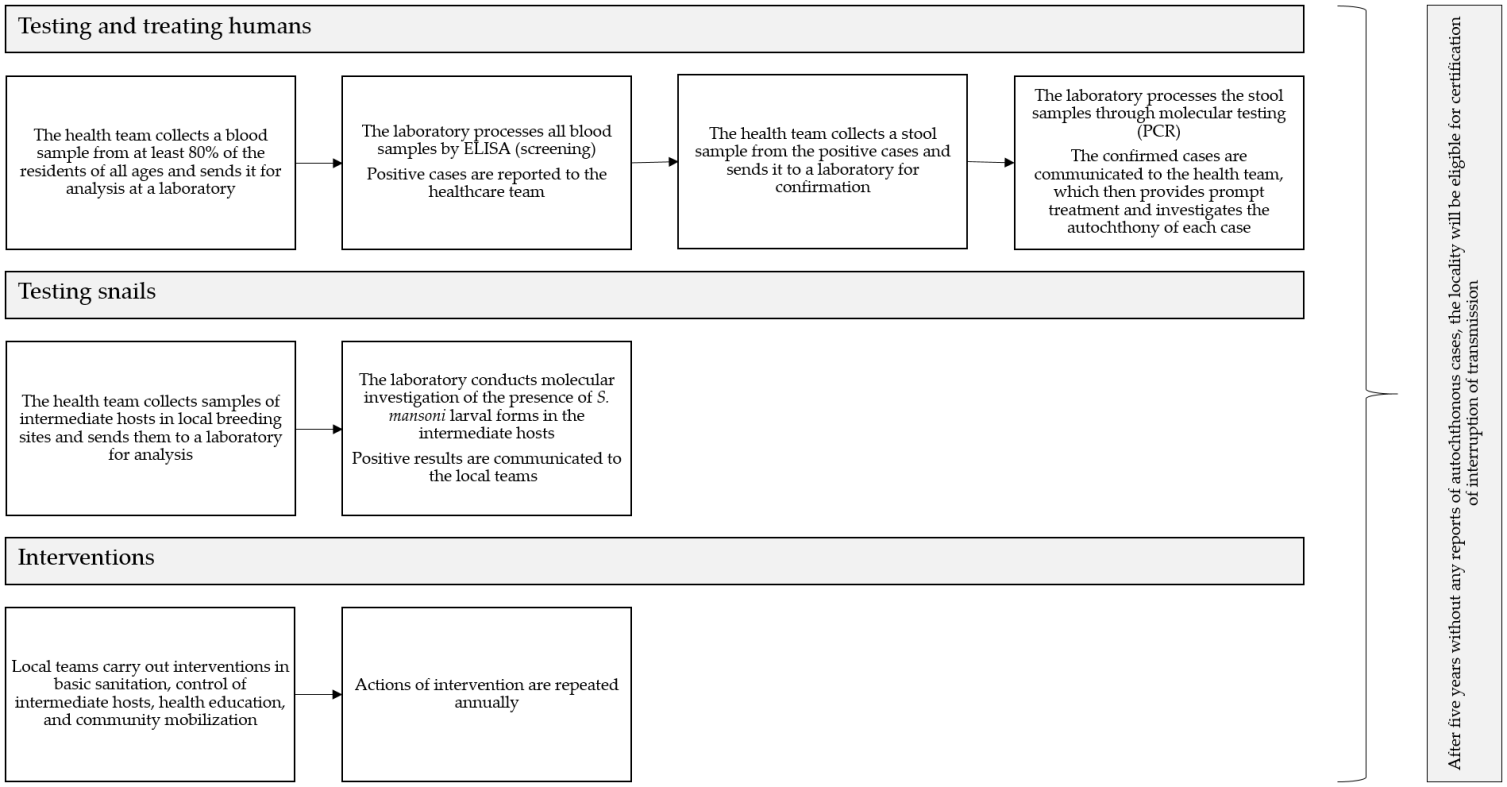
Action plan suggested by FioSchisto to verify interruption of schistosomiasis transmission in eligible locations.

Thus, by implementing the test-and-treat strategy, all individuals with schistosomiasis positivity are identified and treated. To make it possible, it is necessary to determine a new testing workflow so that samples are concluded as negative with the highest possible accuracy after multiple stages of analysis. The flowchart described in **Figure 1** demonstrates the action plan proposed by FioSchisto for verifying the interruption of transmission in eligible locations.

Regarding testing in snails, in each monitoring cycle specific identification of the snails should be performed, in addition to detecting *S. mansoni*. The sampling strategy must consider local weather conditions and snail biology. Monitoring should be carried out annually during the dry season when breeding sites typically harbor a larger number of snails. The collection should be performed by trained teams so that a significant number of snails are collected (at least 20 per point), to increase the chances of detecting the parasite. In areas of low endemicity, where the probability of collecting snails positive for the parasite is low, it is necessary to increase the number of snails collected (100 specimens per point, of varying sizes). It is important to mention that these numbers may vary depending on the snail species. In intermittent watercourses, the collection should be performed when there is still enough water to harbor a dense population of snails. The transmission sites in areas considered epidemiologically important should be periodically monitored. The collection sites should be georeferenced, and the maximum amount of information should be recorded.

For the collection, transport, and processing of mollusks, FioSchisto recommends following the instructions in the “Guidelines for Surveillance and Control of Mollusks of Epidemiological Importance: Technical Guidelines” from the Schistosomiasis Surveillance and Control Program (SISPCE) (53). The identification of mollusks should be done using morphological taxonomy (55) and, when necessary, in association with molecular taxonomy (56, 57). Considering the difficulties described above, FioSchisto recommends that specific legislation be developed for the transportation of biological material that is of public health importance, thus facilitating access to reference laboratories. For the detection of *S. mansoni* in snails in areas of high and medium endemicity, where *B. glabrata* is the transmitting species, the classical parasitological methods of light exposure and crushing the snails between glass plates (58) should be prioritized due to their low cost. Considering trematodes diversity in Brazil, morphological identification of cercaria can provide robust results, particularly in endemic areas (59). In areas where *B. straminea* and *B. tenagophila* are the transmitting species, as well as in low-endemicity areas, regardless of the transmitting species, molecular methods are indicated due to the inefficiency of parasitological methods under such epidemiological conditions. In this case, a pool of at least 10 to 20 snails from the same collection point should be used. DNA extraction should follow the literature recommendations (60) and extraction methods should be performed according to the standardized protocols of each laboratory. It is suggested to use the extracted DNA to identify the snail species and to detect the presence of *S. mansoni* (56). Associated with the monitoring of infected snails, it is recommended to investigate the occurrence of naturally infected wild rodents and other mammalians, and their function as maintainer of transmission of *S. mansoni*.

The WHO criterion for interruption of transmission should be used, i.e., no autochthonous cases reported for five consecutive years. The main operational indicators for mollusk control are the percentage of breeding sites surveyed, which indicates whether monitoring actions are being carried out according to the agreed plan; the percentage of positive snails; and the percentage of active transmission foci. These indicators demonstrate whether sanitation, environmental control, and mollusk control actions are effective in eliminating schistosomiasis transmission foci.

#### 2.4.3 Investment in research

In the case of human testing, it is necessary to perform a laboratory and clinical validation of the serological diagnostic tests for schistosomiasis registered by ANVISA. There is also a need for studies that evaluate the effectiveness of the two-step diagnosis strategy and also of the test-and-treat strategy in reducing the number of infection cases in endemic areas. To drive future research efforts, an action plan was outlined:

- It is suggested to create a multicenter sample biobank of serum, urine, feces, and genetic material to be used in the validation of currently available tests and development of point-of-care tests.
- It is also suggested that in the case of fecal samples, unfixed frozen samples and samples processed *via* Helmintex should be stored.
- The case-finding strategy should also be validated because the higher the surveillance, the lower the prevalence tends to be.
- Equally important is the development of a point-of-care test to be used as the first test in diagnosis. For the production of this test, FioSchisto suggests the prioritization of antigens already used in ELISA assays. In the Brazilian states not yet equipped and capable to perform serological tests for schistosomiasis, it is recommended training and implementation of the required measures.

In the case of testing mollusks, it is necessary to provide financial support for biological material collection and transportation. New alternatives for sending preserved mollusks without the use of liquid fixatives, fixed in alcohol, or previously processed by regional teams, can aid increase the efficiency of monitoring methods.

Among promising monitoring techniques, environmental DNA (eDNA) appears to be of great relevance, as it allows the detection of cercariae without the need for mollusks collection. However, this technique still needs better standardization, and its reliability and reproducibility must be evaluated. Another promising method is near-infrared vibrational spectroscopy (NIR), already used for the detection of Zika virus and other arboviruses. NIR is a rapid method that does not require reagents and has good cost-benefit. An estimate regarding the diagnosis of Zika virus showed that NIR was 18 times faster and 110 times cheaper than RT-qPCR (61). However, the equipment is still not widely available, and the standardization of its use for *S. mansoni* detection and *Biomphalaria* spp. identification should be investigated (62).

## 3 Discussion

The successive WHO guidelines from 1953, 1961, 1965, 1967, 1973, and 1980 for the evaluation and control of schistosomiasis focused on the chemical control of snails until the early 1980s. This focus was gradually replaced by collective treatment until the end of the 1990s. Recently, targeted chemotherapeutic control for the most vulnerable groups has gained prominence. Other control measures, although recommended, did not have as much priority over the decades.

Brazil conducted national prevalence surveys of schistosomiasis since the 1940s and, faced with the expansion of the disease, control programs were created, such as the Special Schistosomiasis Control Program (PECE) in 1975, and the SISPCE (current program), which allowed disease mapping and implementation of control measures nationwide. These measures included coproscopic surveys, epidemiological surveillance of cases, environmental surveillance of intermediate hosts, and measures that precede and accompany all control activities, such as education, health, and community mobilization (2). However, since the beginning of Brazilian control programs, preventive chemotherapy as an isolated measure had already proven to be ineffective in reducing the prevalence of the disease (63), especially in areas that remained refractory to treatment (64).

The great diversity of ecological, socioeconomic, and cultural situations has an impact on the epidemiology of schistosomiasis and the dynamics of *S. mansoni* transmission in Brazil. The disease is not evenly distributed and low endemic areas are predominant, where it is very likely that the proportion of severe infections (≥ 400 epg by Kato-Katz) is less than 1% (32). The reduction in schistosomiasis prevalence and parasite load in infected individuals is the result of control programs that historically have used a combination of approaches, such as mass treatment, molluscicide application, and increased coverage of safe water supply and sanitation (65–67).

Studies conducted in Brazil have shown that periodic mass treatment of people living in endemic communities without improvements in water supply and sanitation infrastructure has a transient effect (3). Regarding molluscicides, although studies have shown that the use of niclosamide is capable of reducing snail populations and producing a substantial short-term impact on the prevalence and incidence of human infection, it has the disadvantage of low cost-effectiveness (27), toxic action on non-target organisms (68) and the need for frequent applications (65, 67). The literature has shown that niclosamide interferes with the development of zebrafish (*Danio rerio*) through various mechanisms (69). Considering that these studies were conducted at environmentally realistic concentrations, the adverse and toxic effects observed in zebrafish, an animal model universally used for toxicological and genetic testing due to its 70% genetic homology with humans, may also affect wildlife and humans (70).

The FioSchisto, as Fiocruz, and the WHO converge on a common premise: diseases are now recognized as resulting from dysfunction within ecosystems, characterized by their complex interactions. Human health is intimately linked to animal and environmental health. With that being said, the position established by FioSchisto in this document presents itself as a collaborative partner in a directed effort towards a comprehensive understanding of the mechanisms that can lead to the control and elimination of these infectious agents. Within this framework, which encompasses diverse and complementary approaches, research on schistosomiasis in Brazil aims to contribute to the development of strategies and affirmative actions, opening dialogue and a different perspective for collective consideration on the elimination of schistosomiasis.

According to this statement, FioSchisto firmly believes in the inappropriateness of WHO recommendations to the Brazilian reality and the need for their adaptation to the Brazilian context. The emphasis on mass administration of praziquantel and chemical control of snails does not apply to Brazil, given the eco-epidemiological context, socioeconomic particularities, public health policies, and history of disease control. In this scenario, it is necessary to identify infection carriers, provide selective treatment, and improve basic sanitation and surveillance, which is considered the main measure to meet the goal of eliminating schistosomiasis.

To identify infection carriers, FioSchisto recommends two-stage immunological and molecular testing (screening and confirmation) to assess the interruption of transmission throughout the intervention period. It is crucial for the Brazilian government to invest in research for the diagnosis of schistosomiasis and, once appropriate methods are developed, to ensure their prompt availability to all healthcare facilities. Furthermore, it is crucial that the healthcare system is adequately prepared to diagnose possible cases imported by travelers from other countries, involving other species of *Schistosoma*, and to identify associated clinical forms that are not observed in Brazil, such as urinary tract disorder and female genital schistosomiasis.

Praziquantel administration should be conducted under medical supervision within primary health care. The WHO recommendation for mass treatment should only be considered as an initial attack strategy in areas with high endemicity, and it should always be integrated with health education, snail control, and sanitation actions. Additionally, would be highly important to test the new pediatric formulation in the Brazilian population to expedite its use.

Comprehensive health and environmental education should be associated with basic sanitation interventions and other control actions, involving the school community, the general population, and health teams. Snail control should prioritize interventions in breeding sites, with modification of habitats through vegetation removal, drainage of flooded areas, lining of irrigation channels, and careful alteration of water flow.

All agencies involved in basic sanitation should work together with primary health care teams for effective planning, execution, monitoring, and evaluation of actions in municipalities considered a priority from an epidemiological perspective. It is also necessary to promote continuing education programs to keep primary healthcare teams up to date on disease surveillance and control strategies, following the current guidelines of the Ministry of Health. To assist decision-making, as well as monitoring and evaluation of strategic actions, there is a need for an information system that allows for the recording, consolidation, and sharing of relevant data.

The main actions of basic sanitation include the supply of safe drinking water, safe sanitation by sewerage or onsite solutions, and hygiene measures, which may also include solid waste management and drainage of rainwater. Adequate interventions in sewage disposal can contribute to introducing a barrier in the disease transmission cycle by eliminating contact between the eggs present in feces and the intermediate host. It is important to highlight that the design of these interventions must consider the local context and measures for sewage treatment and disposal that effectively remove eggs and miracidia, and ensure sustainability in the system operation.

Access to safe sanitation systems helps prevent wastewater from flowing into ditches or stormwater drainage systems. Localities with poor sanitation conditions may have higher transmission rates because they provide ideal conditions for the breeding of snails (71, 72). Countries that have successfully eliminated the infection, such as Japan (50) and Puerto Rico (73), have done so through intense economic development, government-funded projects, effective community participation, and primarily, increased access to basic sanitation interventions, resulting in a reduction in transmission sites (74, 75).

The FioSchisto considers that the recommendations regarding interventions in basic sanitation, which are part of the guidelines of the Ministry of Health (2) remain valid as a priority measure envisioning the elimination of schistosomiasis transmission. Therefore, it is reiterated that interventions in basic sanitation, when reaching levels of salubrity to protect and improve the living conditions of populations, can have a lasting and effective effect on the control of schistosomiasis, even in areas with low prevalence. There is undeniable evidence of its effect also in the control of other water and sewage-related diseases, such as diarrheal diseases, hepatitis A and E, arboviruses (dengue, Zika, and chikungunya), giardiasis, and different helminthiases. Furthermore, efforts to eliminate schistosomiasis in Brazil will only succeed with a shift in paradigm from the vertical, prescriptive framework to a community-centered approach involving strong intersectoral and interdisciplinary collaboration.

## Author contributions

CM: Conceptualization, Methodology, Writing – original draft, Writing – review & editing, Formal Analysis. LM: Conceptualization, Formal Analysis, Methodology, Writing – original draft, Writing – review & editing, Visualization. SJ: Conceptualization, Formal Analysis, Methodology, Writing – original draft, Writing – review & editing. AM: Conceptualization, Methodology, Writing – review & editing, Validation. AD: Conceptualization, Methodology, Writing – review & editing. AJ: Conceptualization, Methodology, Writing – review & editing, Validation. CM: Conceptualization, Methodology, Writing – review & editing. CB: Conceptualization, Methodology, Writing – review & editing. CM: Conceptualization, Methodology, Writing – review & editing. CL: Conceptualization, Methodology, Writing – review & editing. CF: Conceptualization, Methodology, Writing – review & editing. EO: Conceptualization, Methodology, Writing – review & editing. EG: Conceptualization, Methodology, Writing – review & editing. ES: Conceptualization, Methodology, Writing – review & editing. FB: Conceptualization, Methodology, Writing – review & editing. FS: Conceptualization, Methodology, Writing – review & editing. Id: Conceptualization, Methodology, Writing – review & editing. JM: Conceptualization, Methodology, Writing – review & editing. LH: Conceptualization, Methodology, Writing – review & editing. LF: Conceptualization, Methodology, Writing – review & editing. LB: Conceptualization, Methodology, Writing – review & editing. MS: Conceptualization, Methodology, Writing – review & editing. ML: Conceptualization, Methodology, Writing – review & editing. MM: Conceptualization, Methodology, Writing – review & editing. ME: Conceptualization, Methodology, Writing – review & editing. MF: Conceptualization, Methodology, Writing – review & editing. NK: Conceptualization, Methodology, Writing – review & editing. OC: Conceptualization, Methodology, Writing – review & editing. PP: Conceptualization, Methodology, Writing – review & editing. RN: Conceptualization, Methodology, Writing – review & editing. SG: Conceptualization, Methodology, Writing – review & editing. Sd: Conceptualization, Methodology, Writing – review & editing. ST: Conceptualization, Methodology, Writing – review & editing. TF: Conceptualization, Methodology, Writing – review & editing. CG-T: Conceptualization, Investigation, Methodology, Supervision, Validation, Writing – original draft, Writing – review & editing. OP: Conceptualization, Investigation, Methodology, Supervision, Validation, Writing – original draft, Writing – review & editing. RC: Conceptualization, Investigation, Methodology, Supervision, Validation, Writing – original draft, Writing – review & editing. RS: Conceptualization, Investigation, Methodology, Supervision, Validation, Writing – original draft, Writing – review & editing. RR: Conceptualization, Investigation, Methodology, Supervision, Validation, Writing – original draft, Writing – review & editing. RO: Conceptualization, Investigation, Methodology, Supervision, Validation, Writing – original draft, Writing – review & editing.
